# Nitrogen and Boron Co-Doped Graphene Oxide Quantum Dots: Top-Down Fabrication, Comprehensive Characterization and Antibacterial Activity Against Multidrug-Resistant ESKAPE Pathogens

**DOI:** 10.1021/acsomega.6c01888

**Published:** 2026-05-06

**Authors:** Albina Mikhraliieva, Olga Bragina, Yutao Xing, Yevgen Karpichev, Volodymyr Zaitsev

**Affiliations:** 1 Department of Chemistry, Pontifical Catholic University of Rio de Janeiro, Marquês de Sao Vicente Street, 225, Rio de Janeiro, RJ 22451-900, Brazil; 2 Department of Chemistry and Biotechnology, 54561 Tallinn University of Technology, 15 Akadeemia Rd., 12618, Tallinn, Estonia; 3 Laboratório de Microscopia Eletrônica de Alta Resolução, Centro de Caracterização Avançada para a Indústria de Petróleo (LaMAR/CAIPE), 28110 Universidade Federal Fluminense, Niterói, RJ 24210-346, Brazil

## Abstract

Herein, we present the synthesis of three types of graphene oxide quantum dots (GOQDs) having different dopants, namely, nitrogen and boron. Doped GOQDs were successfully synthesized via a one-step top-down hydrothermal treatment of graphene oxide in the presence of H_2_O_2_ and appropriate dopant precursors. The nanoparticles were characterized using TEM, XPS, UV–vis, and photoluminescence spectroscopy. The applied strategy provides a reproducible route to anisotropic nanoparticles with heights in the range of 1.0–1.5 nm and lateral sizes in the range of 7–10 nm, with a well-defined graphenic structure of the core and defined compositional and optical characteristics, highlighting the advantages of top-down approaches over bottom-up methodologies for systematic structure–property investigations. XPS analysis demonstrated nitrogen incorporation into the graphenic lattice of all studied GOQDs, primarily in pyrrolic and graphitic forms, whereas the introduction of boron through boric acid or 3-aminophenylboronic acid predominantly affected edge functionalities. Biological evaluation revealed a pronounced dependence of antibacterial activity on GOQD composition. Among the three materials, nitrogen-doped GOQDs exhibited broad-spectrum bactericidal activity against the *ESKAPE­(E)* panel, with a particularly low MBC of 8 μg/mL against *Staphylococcus aureus*. Boron- and nitrogen-*co*-doped GOQDs exhibited pronounced bacteriostatic activity against *S. aureus* within the concentration range of 100–4 μg/mL. Cytotoxicity assays using HaCaT keratinocytes demonstrated that N-GOQDs combine effective antibacterial performance with excellent cytocompatibility, achieving bacterial eradication at concentrations that remain entirely nontoxic to human skin cells. The results provide clear structure–activity insights and position N-GOQDs as a promising platform for developing next-generation antibacterial nanomaterials targeting multidrug-resistant ESKAPE pathogens.

## Graphical abstract

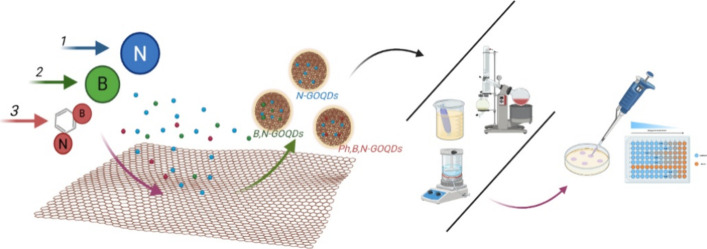

## 1 Introduction

The abuse of antibiotics greatly increases the risk of bacterial resistance. Therefore, it is crucial to develop more efficient antibacterial materials. In this context, nanotechnology emerges as a promising alternative to conventional molecular antibiotics, as nanomaterials can interact with bacterial cells through multiple mechanisms enabled by their nanoscale dimensions, high surface-to-volume ratio, and tunable surface chemistry, thereby significantly reducing the likelihood of resistance development and enhancing antibacterial efficacy.^1^

Antimicrobial resistance (AMR) is a pervasive global health crisis, constituting a fundamental threat to human welfare.^2^ This resistance arises when microbial populations cease to respond to antimicrobial therapeutics, severely complicating infection management. The resultant morbidity and mortality are staggering, with 4.95 million estimated deaths attributed to bacterial AMR in 2019.^3^ The widespread use of antibiotics, self-medication, and exposure to hospital environments collectively drive the proliferation of multidrug-resistant (MDR) bacteria.^4^

In Gram-negative bacteria, the dissemination of extended-spectrum beta-lactamase (ESBL)-producing strains significantly compromised the efficacy of beta-lactam antibiotics (e.g., penicillin, cephalosporins, and monobactams).^5^ Conversely, beta-lactam resistance in Gram-positive bacteria is commonly mediated by alterations in bacterial penicillin-binding proteins (PBPs), the targets of all beta-lactam agents. However, some bacterial groups already resist these “last-resort antibiotics”.^6,7^

The group of pathogens known as ESKAPE (*Enterococcus faecium* (*E. faecium*), *Staphylococcus aureus* (*S. aureus*), *Klebsiella pneumoniae* (*K. pneumoniae*), *Acinetobacter baumannii* (*A. baumannii*), *Pseudomonas aeruginosa* (*P. aeruginosa*), and *Enterobacter cloacae* (*E. cloacae*)) represents clinically important bacterial species that are responsible for most hospital-acquired drug-resistant infections.^8^

Increasing therapeutic failure necessitates the exploration of novel modalities to control infectious diseases.^4^ In this context, nanotechnology emerges as a promising alternative to conventional molecular antibiotics, as nanomaterials can interact with bacterial cells through multiple, nonspecific mechanisms enabled by their nanoscale dimensions, high surface-to-volume ratio, and tunable surface chemistry, thereby significantly reducing the likelihood of resistance development and enhancing antibacterial efficacy.^9,10^ Specifically, nanomedicine strategies employing nanoparticles are of high interest, owing to their capacity as nanostructures to circumvent existing MDR mechanisms, thereby enabling the elimination of pathogens resistant to conventional antibiotic therapy.^11^

Carbon dots (CDs), as a new class of zero-dimensional carbon-containing nanoparticles, have rapidly gained attention due to their high stability, low cytotoxicity,^12^ and strong photoluminescence (PL).^13^ The intense scientific interest is reflected in the dramatic increase in ″carbon dot″ publications over the last 5 years, rising to 5000 per year (according to Scopus data). Significant progress has been made in the application of CDs for diverse biomedical purposes including cellular imaging,^14^ biosensing,^15^ and drug delivery.^16^

Generally, CDs can be divided into two categories depending on the synthetic approach:^17^ (a) CDs obtained in “bottom-up” synthesis, in which nanoparticles are produced from small organic precursors, typically via thermal treatment (inducing polymerization and carbonization), and (b) CDs obtained in “top-down” synthesis, from larger carbonaceous materials. Previously, it was assumed that both methods would yield the same nanoscale materials. However, their significant differences have been demonstrated. CDs obtained in ″top-down″ synthesis always have a graphene core and anisotropic geometry (lateral sizes of nanoparticles can reach 40 nm or more at a height of 1–2 nm). CDs obtained via ″bottom-up″ synthesis exhibit an isotropic geometry and a size below 5 nm. The mechanism of CD formation in the ″top-down″ approach involves cutting graphene 2D sheets into smaller fragments. Therefore, top-down particles are most commonly referred to as graphene (or graphene oxide (GO), depending on the C/O ratio) quantum dots (G­(O)­QDs). The mechanism of CD formation obtained via the ″bottom-up″ approach remains unclear. Depending on the synthetic conditions and chemical composition of the source compounds, CDs can vary in chemical composition, having (or not) a graphene core. According to a key review,^18^ CDs obtained via a bottom-up approach are classified into three groups: carbon polymer dots (CPDs), carbon nanodots (CNDs), and carbon quantum dots (CQDs).

Because CDs can be readily obtained from various precursors by the bottom-up method, their antibacterial properties have been most extensively studied. For example, antibacterial activity of sulfur- and nitrogen-doped CDs (in the article they are called *CQDs*) against *Bacillus subtilis* and *Escherichia coli* (*E. coli*) bacterial strains was studied for the first time in 2018.^19^ It was demonstrated that N-doped *CQDs* caused bacterial death linked to electrostatic interactions with cell membranes, while S-doped *CQDs* showed a size-dependent inhibition of the Gram-positive bacterial growth.^19^ P-doped *CQD*s exhibited similar antibacterial activity.^20^ The minimal inhibitory concentration of *P-CQDs* was 1230 μg/mL (for *E. coli*) and 1440 μg/mL (for *S. aureus*). Boric acid–functionalized CDs prepared via a bottom-up approach exhibited significantly higher antibacterial activity against *E. coli* than against *S. aureus*, attributable to differences in bacterial cell wall architecture.^21^ In 2024, carbon dots derived from onion powder and further functionalized with poly­(hexamethylene biguanide) hydrochloride were reported as effective antibacterial agents against ESKAPE pathogens. The authors attribute the effective inhibition of biofilm formation in both Gram-positive and Gram-negative bacteria by these carbon dots to their ability to induce the generation of reactive oxygen species (ROS).^22^

The studies described above clearly demonstrate the antibacterial potential of CDs. However, the unclear mechanism of CDs formation in the bottom-up approach is a key limitation, resulting in poor control of the nanoparticle chemical composition and morphology, which commonly leads to structural heterogeneity and inconsistent biological outcomes, complicating the systematic understanding of structure–activity relationships. To address these challenges, the present research emphasizes the critical importance of the precursor selected for CD synthesis in the systematization of antimicrobial knowledge. In particular, three samples of GOQDs doped with nitrogen and boron were obtained from graphene oxide via a one-step top-down hydrothermal synthesis, characterized, and assessed for antibacterial activity against the ESKAPE group and *E. coli* (ESKAPE­(E)). This study provides a promising foundation for the development of antibacterial nanodrugs against ESKAPE­(E) pathogens.

## 2 Materials and Methods

### 2.1 Reagents and Solutions

Graphite was purchased from Synth (Brazil). Potassium permanganate (KMnO_4_, ≥99.0%), hydrogen peroxide solution (H_2_O_2_, 30% (w/w)), ammonia solution (25%), sodium chloride (99.5%), sulfuric acid (95–98%), boric acid (95%), acetic acid (95%), and phosphoric acid (85%) were purchased from Sigma-Aldrich. (3-Aminophenyl)­boronic acid (APBA, 98%) was supplied from AmBeed (https://www.ambeed.com).

All aqueous solutions were prepared using ultrapure water obtained from the PURELAB Classic water-purification system (Elga, U.K.). Dialysis tubes from Spectrum Lab with pore sizes of 500 Da and 6000–8000 Da were used for nanoparticle purification.

### 2.2 Instrumentation

An Amicon stirred cell with a hydrophilic 0.45 μm PTFE (11806-47N, Sartorius) was used to separate GOQDs and residual components after synthesis. The pH of the solutions was measured using a PHS-3E (China) meter with a BioTrode (Hamilton, USA) electrode. UV–visible (UV–vis) absorption spectra in the 200–700 nm range were measured on a Cary 60 UV–visible spectrophotometer from Agilent (USA). Photoluminescence (PL) measurements were performed using an RF-6000 spectrofluorometer (Shimadzu, Japan) with 1 cm optical path-length quartz cuvettes. The spectra were analyzed using Spectragryph 1.2.121 and OriginPro 2021. Atomic force microscopy (AFM) topography images were obtained in a MultiMode8 atomic force microscope (Bruker) in Peak Force Tapping mode. A ScanAsyst-Air probe (Bruker) with a spring constant of 0.4 N/m and a 2.0 nm tip end radius was used for the scanning. The AFM platform was mounted in an environmental chamber that allows control of relative humidity and temperature (10% and 25 °C). For AFM measurements, the samples of GOQDs were diluted in ultrapure water, and a single drop of the suspension was added to a clean mica and then dried in a nitrogen flux for 2 h. The statistical analysis was performed using Gwyddion 2.53 software. The size and morphology of nanoparticles were investigated using transmission electron microscopy (TEM) and high-resolution (HR) TEM on a JEOL JEM-2100F operated at 200 kV with a field-emission gun. The TEM samples were prepared by drop-casting suspensions of GOQDs onto an ultrathin carbon-coated copper grid and then drying in air. The surface composition of the samples was determined by XPS using a Kα X-ray photoelectron spectrometer from Thermo Fisher Scientific, equipped with a hemispherical electron analyzer and an aluminum anode X-ray source (*K*α = 1486.6 eV), providing an energy resolution of ≈1 eV.

### 2.3 Biomedical Assays

The clinical isolates *S. aureus* HUMB 19594, *E. faecium* HUMB 65620, *K. pneumoniae* HUMB 01336, and *P. aeruginosa* HUMB 4438 D01–10 were obtained from the Human Microbiome Project culture collection (HUMB; https://eemb.ut.ee). *E. coli* DSM 500, *E. cloacae* DSM 109592, and *A. baumannii* DSM 25645 were obtained from the German Collection of Microorganisms and Cell Cultures (DSMZ).

#### Minimal Bactericidal Concentration (MBC)

GOQD samples were diluted in water to a final concentration of 1000 μg/mL. For antibacterial evaluation, all prepared samples were subjected to serial dilutions across the tested bacterial strains at concentrations ranging from 4 to 500 μg/mL. In parallel, MBC values were determined for the reference antibiotics amoxicillin, polymyxin B, and daptomycin using the same assay format. Stock solutions of each antibiotic were prepared at an initial concentration of 250 μg/mL, and serial dilutions were made prior to testing.

A single colony grown on a Tryptic Soy Agar (TSA agar) plate was inoculated into TSB broth and incubated for 16 h at 37 °C with agitation at 150 rpm. The overnight culture was subsequently diluted 1:50 in fresh medium and further cultivated under the same conditions until reaching the exponential growth phase (OD_600_ = 0.6). Cells were harvested by centrifugation at 5000*g* for 5 min, after which the pellet was resuspended in an equal volume of sterile water. The suspension was then adjusted with sterile water to achieve a final cell density of OD_600_ = 0.2.

For the MBC assay, 90 μL of the bacterial suspension was mixed with 90 μL of the diluted compound solution and incubated at 37 °C for 24 h. Following the exposure period, 3 μL aliquots of each mixture were drop-plated onto TSA agar and incubated for an additional 24 h at 37 °C. MBC was defined as the lowest concentration of the compound at which no visible colonies were observed in the 3 μL spot. All MBC measurements were performed in triplicate.

#### Cell Viability Assay

The human keratinocyte cell line HaCat (immortalized keratinocytes; ATCC PCS-200-011) was cultured in Dulbecco’s Modified Eagle’s Medium containing 4.5 g/L glucose, l-glutamine, and sodium pyruvate (DMEM, Corning), supplemented with 10% fetal bovine serum (FBS) and 1% penicillin–streptomycin (PEST). Cells were seeded into 96-well plates at a density of 1 × 10^5^ cells per well, using a Countess Automated Cell Counter (Invitrogen), and incubated overnight at 37 °C in a humidified atmosphere with 5% CO_2_.

After 24 h, 100 μL of fresh medium and 10 μL of suspension of doped GOQDs were added to the wells, followed by an additional 24 h of incubation. Wells receiving 10 μL of sterile H_2_O served as solvent controls.

Cell viability was quantified using the WST-1 assay (Roche), which measures mitochondrial dehydrogenase activity by reducing the tetrazolium salt to a water-soluble formazan dye. The number of formats that are formed correlates directly with the number of metabolically active cells. At 24 h post-treatment, 5 μL of WST-1 reagent was added to each well, and plates were incubated for 2 h at 37 °C. Absorbance was subsequently recorded at 450 nm using a TECAN GENios Pro microplate reader (Switzerland).

### 2.4 Synthesis

GOQDs were prepared via a top-down approach by etching GO with H_2_O_2_ in the presence of various additives (dopants), as previously reported.^23,24^

#### Synthesis of N-GOQDs

40 mg of GO was sonicated in 20 mL of water for 60 min and transferred to a Teflon cylinder reactor containing 1.5 mL of NH_4_OH (25%) and 3.0 mL of H_2_O_2_ (30%) in 60 mL of water. The reactor was tightly sealed, heated to 180 °C for 4 h, and then cooled naturally, yielding brown suspensions containing N-GOQDs with a small amount of dark precipitate.

A sample of B,N-GOQDs was obtained similarly to N-GOQDs by adding 0.197 g of H_3_BO_3_ to the GO suspension prior to sonication, yielding a small amount of black fine precipitate and a yellowish liquid.

A sample of Ph,B,N-GOQDs was obtained similarly to N-GOQDs by adding 0.199 g of APBA to the GO suspension prior to sonication, yielding a small amount of sponge-like black solid and a bright-yellow liquid.

##### Postsynthetic Treatment and Purification

First, the suspensions of raw N-GOQDs, B,N-GOQDs, and Ph,B,N-GOQDs were separated from the precipitates by microfiltration using an Amicon aging cell with a hydrophilic PTFE 0.45 μm filter. Second, the suspensions were evaporated under vacuum until a total volume of 20 mL.

The brown suspension of N-GOQDs was purified by adding crystalline NaCl in an amount sufficient to obtain a 0.9% solution, stirring on a vortex until complete dissolution of NaCl, and then incubating for 12 h at 4 °C. As a result of such treatment, orange-yellowish supernatant of *N-GOQDs* was separated from black precipitate of *N-MHG* ^25^ by centrifugation (3600 rpm for 5 min). Finally, water suspension of *N-GOQDs* was purified from molecular impurities (NaCl, NH_3_, H_2_O_2_) by dialysis, using a 6-8 kDa membrane for 4 days. The purification process was monitored by measuring the solution’s electroconductivity. The purification process was considered completed when the resistivity reached 10 MΩ cm. The content of the dialysis bag was evaporated under vacuum, yielding 5 mg (12.5%) of fully dispersible N-GOQDs. The stock suspension of N-GOQDs was obtained by ultrasonication of the sample in ultrapure water to reach a concentration of 400 μg/mL and stored at 4 °C.

The light-yellowish suspension of *B,N-GOQDs* was purified from molecular impurities by dialysis in a 500 Da membrane for 2 days. The purification control was the same as for *N-GOQDs.* The contents of the dialysis bag were evaporated under vacuum, yielding 10 mg (25%) of *B,N-GOQDs.* The stock suspension of B,N-GOQDs was obtained by ultrasonication of the sample in ultrapure water to reach a concentration of 400 μg/mL and stored at 4 °C.

The bright-yellow suspension of *Ph,B,N-GOQDs* was purified and dried as for B,N-GOQDs, yielding 37.5 mg (93.4%) of Ph,B,N-GOQDs, which were redispersed in ultrapure water to prepare a stock solution at 1500 μg/mL.

## 3 Results and Discussion

### 3.1 Synthesis and Purification of Nanoparticles

To produce carbon dots, we deliberately selected a top-down synthetic strategy as it offers superior reproducibility and greater predictability compared with bottom-up approaches (Figure 1). In this method, zero-dimensional graphene oxide quantum dots (GOQDs) are generated by the controlled oxidative cutting of well-defined two-dimensional graphene oxide sheets. Hydrogen peroxide was employed as a mild and controllable cutting agent under hydrothermal conditions, enabling gradual cleavage of the GO lattice along defect sites and oxygen-rich regions.^26,27^ As a result, the formation of GOQDs predominantly follows a physical downsizing mechanism, yielding nanoparticles that inherit structural motifs from the parent GO including graphenic domains and oxygen-containing functional groups. The introduction of NH_4_OH during synthesis further enables nitrogen incorporation via well-established reactions with epoxide, carbonyl, and carboxyl functionalities located on both basal planes and sheet edges.^28^ Such a synthesis pathway contrasts with bottom-up routes, in which nanoparticle nucleation, growth, and surface chemistry are governed by complex, often poorly defined, polymerization and carbonization processes. In the present top-down approach, both the cutting and doping processes are chemically intuitive and mechanistically transparent, which facilitates systematic control over particle size, morphology, and surface composition. Consequently, the resulting GOQDs constitute a more chemically and structurally predictable class of carbon dots, providing a rational platform for correlating synthesis parameters with physicochemical properties and biological activity.

**Figure 1:**
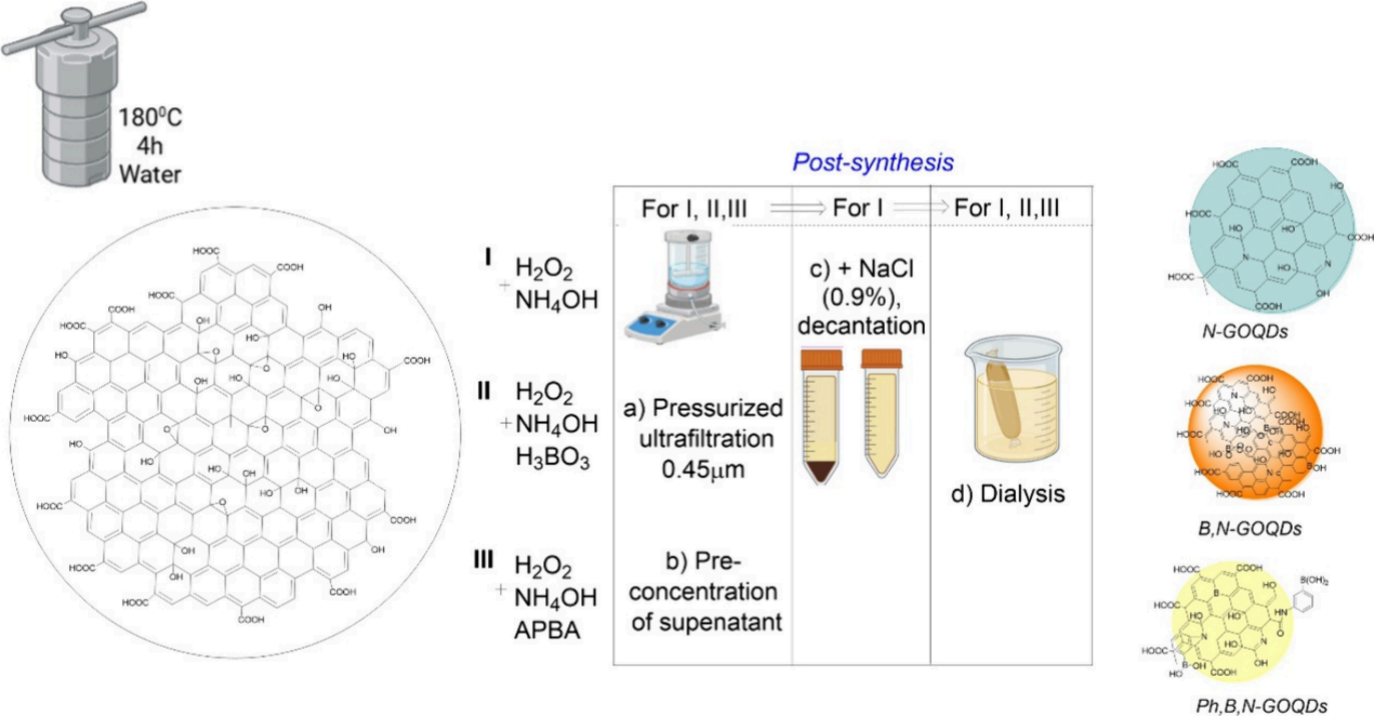
Scheme of the synthetic procedure and obtained nano-objects.

To introduce boron into the graphene oxide quantum dots, two chemically distinct boron-containing additives were employed, namely, boric acid (H_3_BO_3_) and 3-aminophenylboronic acid (APBA). Similar to ammonia, boric acid is capable of interacting both with the graphenic core and with oxygen-containing functional groups located at the edges and basal planes of graphene oxide, predominantly through the formation of B–O–C and borate ester-type linkages.^29^

### 3.2 Chemical Composition

The chemical composition of GOQDs was determined by using XPS analysis. The XPS survey spectra reveal significant variations in the elemental composition of the particles depending on the precursor used (Table 1).

**Table 1:** Elemental Composition and Atomic Ratios of GOQDs Obtained from XPS Survey Spectra

	Composition of nanoparticles, at%					Atomic ratio	
Samples	C 1s	N 1s	O 1s	B	Na	C/O	C/N
N-GOQDs	61.8	4.9	30.0	0.0	3.3	2.10	12.6
B,N-GOQDs	39.6	8.1	45.5	3.3	3.5	0.87	4.90
Ph,B,N-GOQDs	54.2	10.7	33.1	1.1	0.90	1.60	5.00

N-GOQDs consist of 61.8 at. % carbon, 30.0 at. % oxygen, and 4.9 at. % nitrogen, with characteristic binding energies centered at 286.1 eV (C 1s), 531.5 eV (O 1s), and 399.5 eV (N 1s), respectively. Relatively to N-GOQDs, Ph,B,N-GOQDs show a moderate decrease in carbon content (by 12%) and an increase in oxygen content (by 9%), together with a more than 2-fold increase in nitrogen content to 10.7 at. %. (Table 1). B,N-GOQDs, in turn, are characterized by a substantially higher oxygen content (45.5 at. %) and moderately increased nitrogen content (8.1 at. %).

Nitrogen doping of GOQDs is quite aggressive and commonly results in about 5 at. % of nitrogen[Fn fn1], which incorporates into the graphenic core in the form of pyrrolic, pyridinic, or quaternary nitrogen species.^30^ In contrast to alkaline nitrogen doping, boric acid exerts a considerably milder influence on the oxidative cutting of graphene oxide. This reduced reactivity is reflected in both the elemental composition and the synthetic outcome: B,N-GOQDs were obtained in higher yield than N-GOQDs but with moderate boron incorporation (currently 3.3 at. %). A further reduction in oxidative cutting activation was observed when APBA was used as the boron precursor. Consequently, APBA-derived Ph,B,N-GOQDs contain the lowest boron content (1.1 at. %). This behavior can be attributed to the relatively stable aryl-boronic acid environment in APBA, which limits the availability of boron for incorporation into the graphene framework. Notably, the introduction of boron not only modulates the oxidation dynamics but also promotes enhanced nitrogen doping of the GOQDs core. It can be assumed that by partially suppressing excessive lattice degradation and stabilizing oxygen-related defect sites, boron-containing species create a more favorable environment for the incorporation of nitrogen into the graphenic lattice, resulting in experimentally observed higher nitrogen contents compared to solely nitrogen-doped systems.

To gain insights into the origin of these compositional changes, we proceeded with a detailed analysis of the high-resolution XPS spectra. The high-resolution C 1s spectra of GOQDs elucidate the evolution of functional groups across samples. Table 2 presents a detailed analysis of C 1s deconvolution, including the relative contributions of different groups and the possible group types, as a function of binding energy.

**Table 2:** XPS C 1s Fitting Parameters for GOQD Materials

Samples		N-GOQDs		B,N-GOQDs		Ph,B,N-GOQDs	
Peak (eV)	Type	Area ratio	Relative peak area (%)	Area ratio	Relative peak area (%)	Area ratio	Relative peak area (%)
285.0	C–C/C=C	1	69	1	68	1	45
286.5	C–N/C–O	0.14	9	0.23	15	0.53	24
287.8	C=O	0.05	4	0.06	5	0.18	8
288.6	HO–C=O/N–C=O	0.26	18	<0.02	<1	0.24	11
289.2	–O–C=O	0	0	0.16	11	0.26	12

The relative area of the primary C 1s peak at 285.0 eV (sp^2^/sp^3^ carbon in the GOQD core) remains similar for N-GOQDs and B,N-GOQDs (68–69%) but decreases to 45% for Ph,B,N-GOQDs, indicating a major structural modification in the latter (Figure 2). The peak at 286.5 eV (C–O/C–N) increases from 9% (N-GOQDs) to 15% (B,N-GOQDs) and further to 24% (Ph,B,N-GOQDs). The relative area of the 287.8 eV peak (C=O) remains approximately constant across samples. Importantly, the high-energy O–C=O component associated with free carboxyl groups at 288.6 eV markedly decreases in B,N-GOQDs (Table 2).

**Figure 2:**
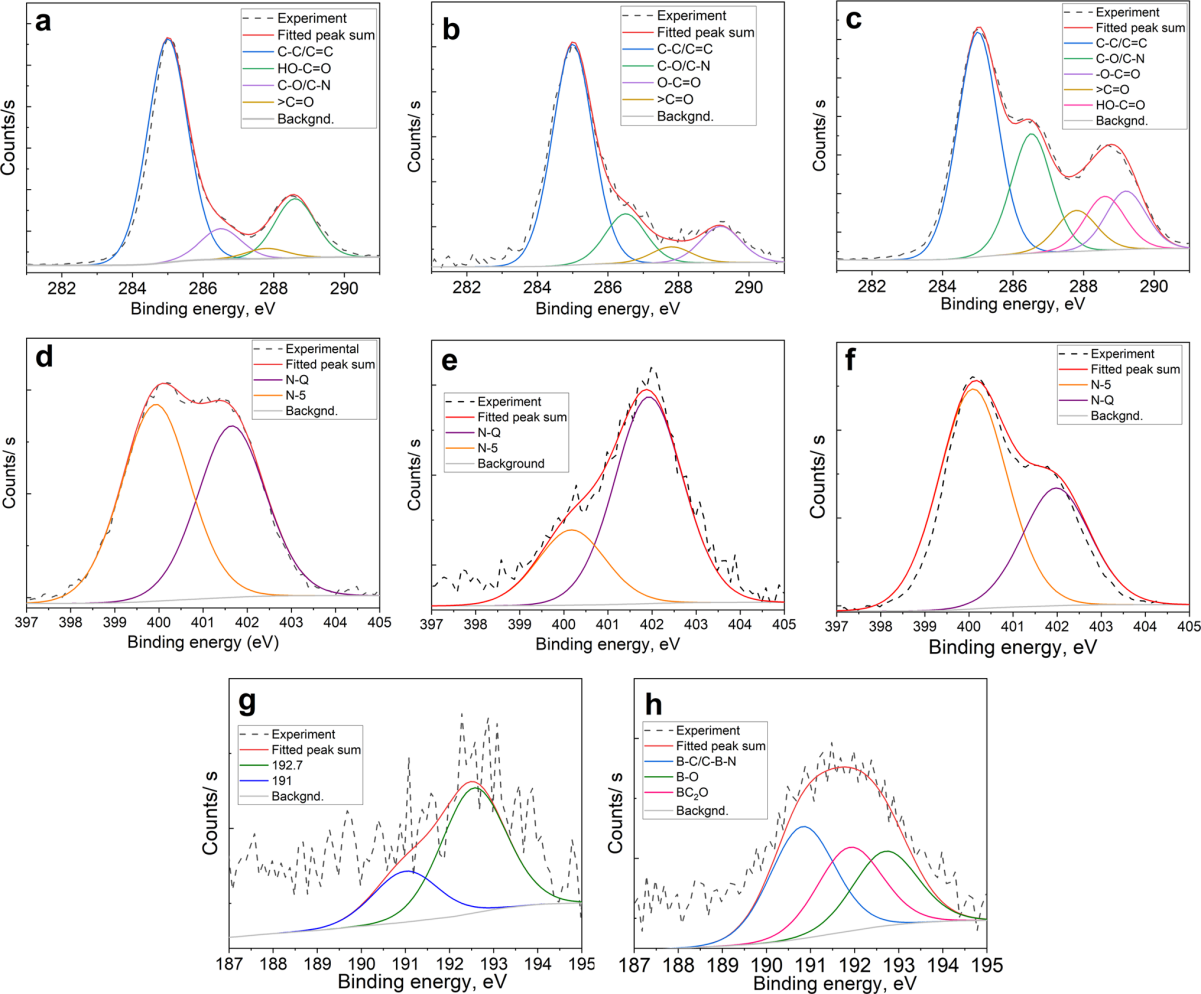
Fitted XPS spectra of the C 1s (a–c), N 1s (d–f), and B 1s (g, h) regions corresponding to N-GOQDs (a, d), B,N-GOQDs (b, e, g), and Ph,B,N-GOQDs (c, f, h).

Combining the XPS survey evidence of increased oxygen content in B,N-GOQDs with the results observed for the C 1s spectrum (namely, constant 285.0 eV core fraction for N-GOQDs vs B,N-GOQDs, growth of C–O/C–N, and the reduction of free carboxyl groups for B,N-GOQDs), we infer that boron predominantly binds via reaction of boric acid with phenolic and peripheral carboxyl groups, forming borate esters. The presence of ester-type O–C=O environments is evidenced by the band at 289.2 eV, which we attribute to esterified O–C=O rather than free −COOH (Table 2).

The nitrogen N 1s environment also shows a clear transition between samples (Figure 2d–f). *N-GOQDs* exhibit a balanced ratio of pyrrolic (N-5, at 400.0 eV) and graphitic nitrogen (N-Q, at 401.5 eV) in graphenic core.^28^ B,N-GOQDs dominate (about 73%) graphitic nitrogen, while in *Ph,B,N-GOQDs*, pyrrolic nitrogen consists of about 65% (Figure 2d–f).

The increase in the relative intensity of the graphitic nitrogen, together with notable increases of nitrogen content in B,N-GOQDs (8.1 vs 4.9 at. % for N-GOQDs), suggests that boric acid moderates the extent of GO lattice degradation by H_2_O_2_, thereby facilitating nitrogen incorporation into the graphene core. Conversely, *Ph,B,N-GOQDs* exhibit a dominant peak at 400.1 eV, corresponding to pyrrolic and amide fragments originating from APBA. This transition suggests that while boric acid forces nitrogen into the internal lattice, the organoboron precursor promotes the retention of active amino-functionalized edge states.

Analysis of the B 1s spectrum highlights distinct chemical states of the boron dopants (Figure 2g,h). In B 1s, XPS spectra of B,N-GOQDs dominate a signal centered near 192.7 eV, indicating the presence of an oxygen environment.^31^ On the contrary, Ph,B,N-GOQD samples exhibit a symmetric peak centered at approximately 192.0 eV, indicating the presence of B–C bonds with a binding energy of 190.9 eV originating from the APBA^32^ (Figure 2h).

### 3.3 Morphological Properties

Atomic force microscopy and transmission electron microscopy were used to characterize the morphology of the synthesized nanoparticles. The AFM analysis reveals a pronounced influence of heteroatom doping on particle dimensions. AFM data reveal a clear influence of the dopants on particle dimensions. The N-GOQDs exhibit a height of approximately 1.5 nm, whereas B,N-GOQDs and Ph,B,N-GOQDs display a reduced height of approximately 1.0 nm together with a decreased lateral size (Figure 3). These morphological differences can be attributed to the structural role of boron-containing dopants during the oxidative cutting of the parent graphene oxide sheets, as demonstrated in Section 3.2. TEM analysis reveals that all three samples N-GOQDs, B,N-GOQDs, and Ph,B,N-GOQDs possess a well-defined crystalline structure with graphenic domains (Figure 3). The measured interplanar spacing of approximately 0.20 nm corresponds to the (101) planes of graphite, confirming the preservation of a graphene-like lattice at the nanoscale. While N-GOQDs and B,N-GOQDs exhibit high image contrast and clearly resolved lattice fringes, Ph,B, N-GOQDs appear as lower-contrast fragments. Also, it is important to mention that the lateral size of the doped nanoparticles decreases with doping from 8 to 10 nm for N-GOQDs to 6–8 nm for B,N-GOQDs and further to 4–6 nm for Ph,B,N-GOQDs. Importantly, these morphological observations are in excellent agreement with the chemical information obtained from XPS analysis. Despite the relatively high oxygen content detected in B,N-GOQDs, the preservation of well-defined graphenic domains observed by TEM confirms that boron doping predominantly affects peripheral functional groups rather than the graphenic core, most likely through the formation of borate ester–type linkages with edge carboxyl and phenolic groups, without disrupting the sp^2^-conjugated lattice. At the same time, nitrogen is incorporated into the graphene framework primarily as graphitic nitrogen, which is structurally compatible with the hexagonal carbon lattice. In the case of GOQDs doped with APBA, the graphenic core undergoes more pronounced structural modifications, indicating a stronger perturbation of the graphene lattice compared to N-GOQDs and B,N-GOQDs.

**Figure 3:**
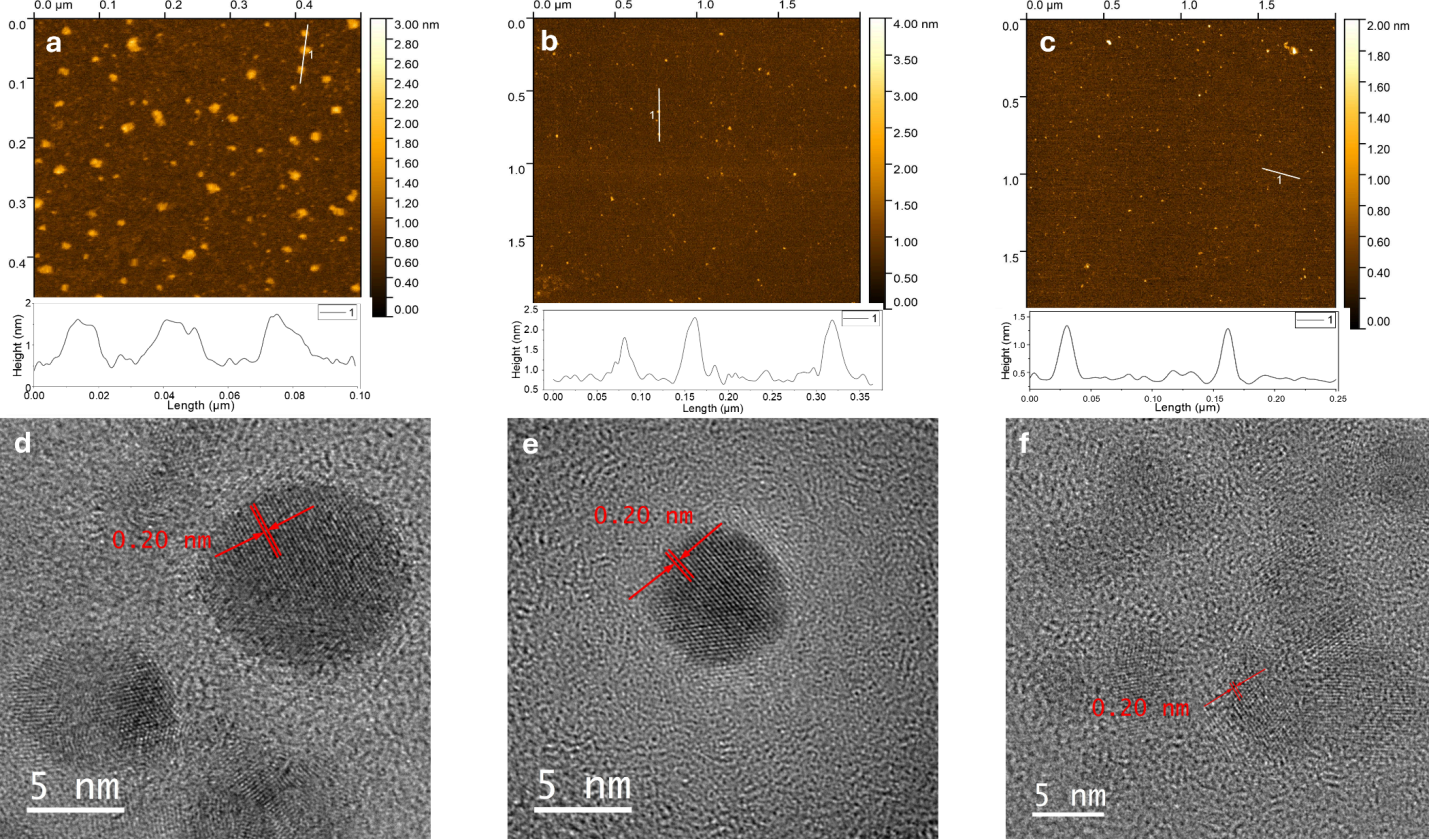
AFM images with depth profiles (a–c) and TEM images (d–f) of N-GOQDs (a, d); B,N-GOQDs (b, e) and Ph, B,N-GOQDs (c, f).

When combined with AFM data, which indicate particle heights in the range of 1.0–1.5 nm, the TEM-derived lateral sizes (7–10 nm) yield an aspect ratio of 6–7. This pronounced anisotropy provides strong evidence that the nanoparticles belong to the class of GOQDs and have planar morphology (in contrast to CDs obtained via top-down synthesis).

### 3.4 Optical Properties

Figure 4a displays the UV–vis absorption spectra for the *N-GOQD*, *B,N-GOQDs*, and *Ph,B,N-GOQDs* over the wavelength range of 200 to 700 nm. Crucially, the spectra are not identical. All three samples exhibit strong absorption in the ultraviolet region below 300 nm, with no detectable absorption above 500 nm. The high absorbance near 230 and 250 can be characteristic of π–π* transitions of the aromatic C=C bonds in the GOQD structure. The distinct shoulder or broader peak in the 260–300 nm region for all samples is typically attributed to the *n–*π* transition of surface-bound oxygen or nitrogen-containing functional groups (C=O, C–OH, and C–N). The solution of APBA exhibited an absorption spectrum in the UV range with two peaks: 234 and 300 nm.^33^ *Ph,B,N-GOQDs* exhibit peaks at 256, 325, and 364 nm, indicating APBA transformation or incorporation onto *Ph,B,N-GOQDs*.^33^ The concentration-dependent UV–Vis extinction reveals clear differences among the three GOQD samples. The suspensions exhibit distinct extinction slopes of 11.8 × 10^–2^ (N-GOQDs), 3.3 × 10^–2^ (B,N-GOQDs), and 2.2 × 10^–2^ (Ph,B,N-GOQDs), reflecting intrinsic variations in their electronic structure and surface chemistry.^34^ These differences confirm that the applied synthesis routes yield chemically distinct nano-objects.

**Figure 4:**
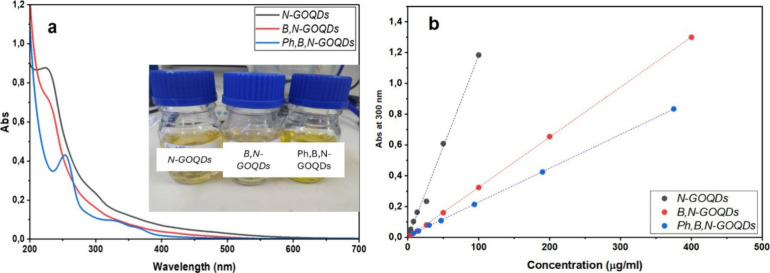
(a) UV–vis spectra of N-GOQDs (black line), B,N-GOQDs (red line), and Ph,B,N-GOQDs (blue line). (b) Corresponding calibration curves. Inset: digital photos of the samples.

The PL spectra of doped GOQDs in aqueous suspensions exhibit pronounced differences not only in the emission maxima but also in the intensity of the excitation-dependent emission (Figure 5). For N-GOQDs, variation of the excitation wavelength from 390 to 470 nm results in a gradual red shift of the PL maximum from 490 to 525 nm without noticeable changes in the emission intensity. Excitation at 430 nm yields the highest PL intensity for N-GOQDs with an emission maximum at 500 nm. In contrast, B,N-GOQDs and Ph,B,N-GOQDs exhibit their strongest emission at 450 and 435 nm, respectively, upon 330 nm excitation. This behavior reflects a stronger excitation-selective activation of emissive states in boron-containing samples. Such excitation-dependent photoluminescence, manifested in both spectral position and intensity variations, is commonly attributed to contributions from multiple emissive centers, including size-dependent quantum confinement, heterogeneous graphitic domains, and diverse surface and edge functional groups.^35^ In this context, the reduced excitation-wavelength dependence of PL intensity observed for N-GOQDs suggests a higher degree of electronic homogenization, likely promoted by the incorporation of nitrogen into the graphenic lattice.

**Figure 5:**
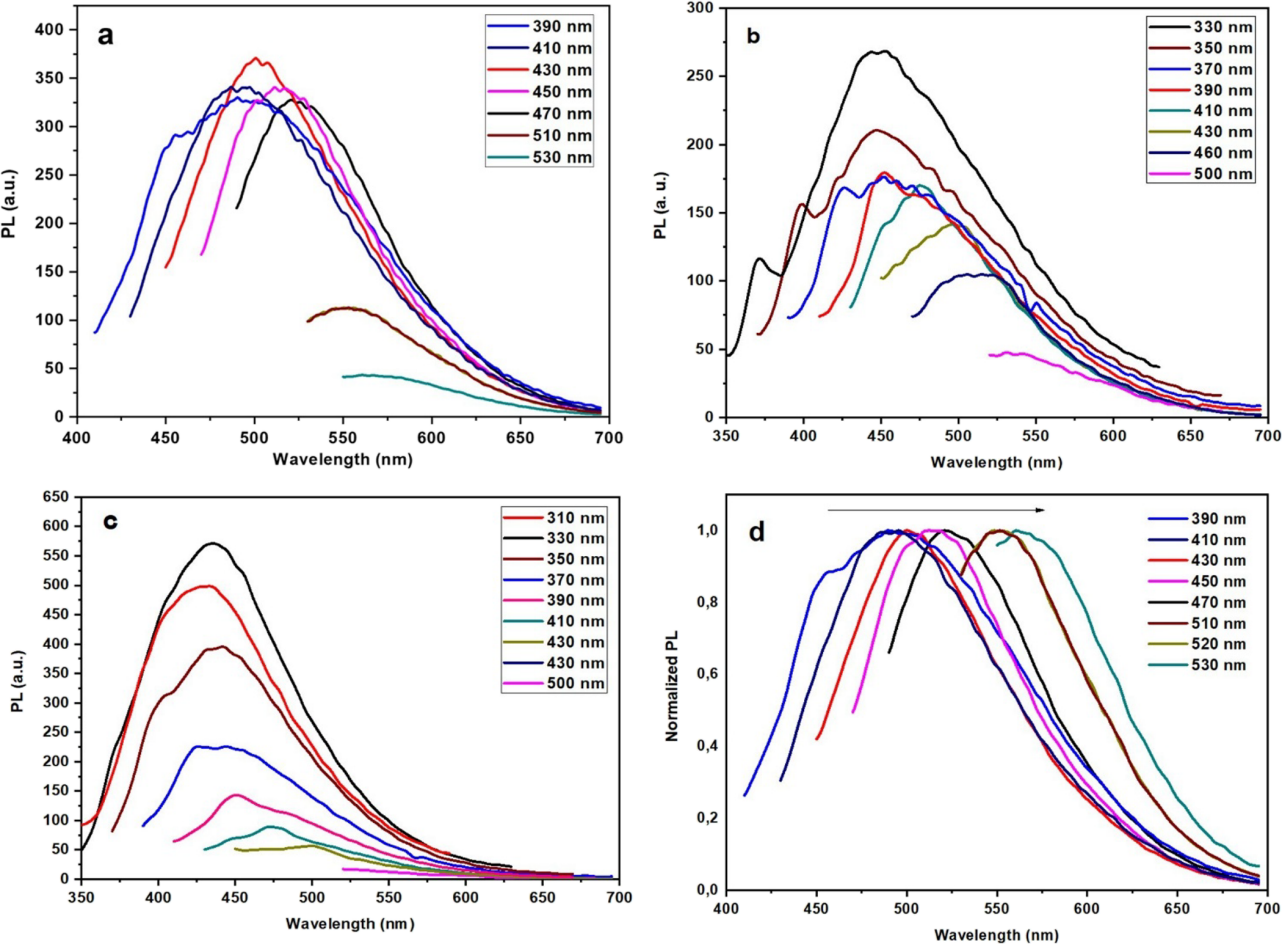
Absolute emission spectra of (a) N-GOQDs, (b) B,N-GOQDs, (c) Ph,B,N-GOQDs, and normalized (d) emission spectra of N-GOQDs under different excitation wavelengths.

Overall, the optical trends are in good agreement with the XPS analysis. The transition from the oxygen-rich border to structurally incorporated N-Q motifs observed in B,N-GOQDs and from amino-enriched edge states in Ph,B,N-GOQDs correlates directly with both the progressive PL blue shift and the enhanced excitation-dependent intensity variations. Together, these results demonstrate that heteroatom doping not only tunes the emission wavelength but also governs the density and distribution of emissive states in GOQDs.

### 3.5 Antibacterial Activity

The antibacterial activity of the doped GOQDs was assessed using six bacterial strains and compared with referenced antibiotics (amoxicillin, polymyxin B, and daptomycin) in the same experimental setup (Table 3). The strains were selected for their critical role in causing hospital-acquired infections worldwide and their frequent resistance to multiple antibiotic classes.^36^ *S. aureus* and *E. faecium* represent Gram-positive bacteria, while the remaining strains serve as models for Gram-negative microorganisms. The antibacterial activities of the doped GOQDs varied among the tested bacterial strains. The resulting MBC values are presented in Table 3.

**Table 3:** MBC Values for Doped GOQDs and Antibiotics (μg/mL)

Sample	S. aureus	E. faecium	K. pneumoniae	P. aeruginosa	A. baumannii	E. cloacae	E. coli
N-GOQDs	8	500	500	500	250	500	500
B,N-GOQDs	4–62^a^	nd^b^	nd	nd	nd	nd	nd
Ph,B,N-GOQDs	4–125^a^	nd	nd	nd	nd	nd	nd
Amoxicillin	0.98	nd^a^	nd	nd	nd	nd	31.2
Polymyxin B	nd	nd	1.95	1.95	1.95	3.9	lethal^c^
Daptomycin	nd	nd	nd	nd	nd	nd	nd

a At the mentioned concentration range, the nanoparticles demonstrate bacteriostatic properties.

b No visible antibacterial activity was detected within the tested concentration range.

c All tested dilutions were bactericidal.

The obtained MBC values indicate a clear difference in bactericidal activity among the doped GOQDs. The N-GOQDs exhibited measurable bactericidal activity against all tested strains of ESKAPE, with high susceptibility to *S. aureus*, for which the MBC reached 8 μg/mL, which is significantly lower than those reported for many carbon dot systems. For example, P-doped carbon nanodots exhibited antibacterial activity with MIC values of 1230–1440 μg/mL against *E. coli* and *S. aureus*,^20^ indicating substantially weaker antibacterial performance. Similarly, S- and N-doped carbon dots reported by Travlou et al. showed antibacterial effects against *E. coli* primarily at concentrations 32 μg/mL.^19^ In contrast, the N-GOQDs in this study achieve bactericidal activity at considerably lower concentrations for *S. aureus*, indicating enhanced potency. For multidrug-resistant ESKAPE­(E) pathogens, recently developed guanidinium-functionalized carbon dots showed effective antibacterial activity; however, their reported MIC concentrations in the range of 3.75 and 5 μg/mL for Gram-positive bacteria to 7.5–15 μg/mL for Gram-negative bacteria depend on the strain and experimental conditions.^22^

Within the 4–125 μg/mL range, boron-*co*-doped GOQDs displayed pronounced bacteriostatic activity toward *S. aureus*, whereas at higher concentrations, the antibacterial response appeared reduced (Figure S1 in the Supporting Information). The observed decrease in antibacterial activity with increasing particle concentration can be explained by their aggregation.^37^ A similar concentration-dependent antibacterial behavior has been reported for other carbon quantum dots. For example, it was suggested that N-doped CQDs exhibited stronger antibacterial effects at lower concentrations, while at higher concentrations, the activity decreased due to nanoparticle aggregation, which limited effective interactions with bacterial cells.^19^ This effect can likewise be attributed to aggregation-induced reduction of available active surface area, highlighting the key role of colloidal stability in antibacterial performance. Indeed, according to XPS results, B,N-GOQDs have a much higher graphitic nitrogen content (6.0%) than N-GOQDs (2.2%)[Fn fn2]. This fact, together with a decrease in the carboxyl group loading at the periphery of boron-containing nanoparticles (Section 3.2), can lead to fever stability of B,N-GOQDs and thus their aggregations, particularly in solutions with high ionic strength. The aggregation of nanoparticles reduces their effective surface area and limits direct contact with bacterial cells, thereby reducing the apparent bactericidal effect despite the higher nominal concentration. Upon serial dilution, the aggregates disperse, exposing more reactive sites and enhancing ROS-mediated oxidative stress on bacterial cells. This phenomenon can account for the paradoxical observation that lower concentrations exhibit stronger antibacterial effects. Similar mechanisms have been reported for other carbon-based nanomaterials, in which ROS-induced damage is considered a primary antibacterial pathway alongside physical contact and membrane disruption.^37^ This pronounced discrepancy highlights the strong dependence of antibacterial efficacy on the physicochemical properties of N-GOQDs, which are determined by synthesis conditions such as precursor composition, reaction temperature, and oxidation level. Variations in surface functional groups, particle size, and defect density likely influenced the interaction between GOQDs and bacterial cells, affecting their ability to induce membrane damage, oxidative stress, or other bactericidal mechanisms.

### 3.6 Cell Viability Assay

The results of the cytotoxicity evaluation of the three GOQD formulations on HaCat keratinocytes are shown in Figure 6. The cytotoxicity assessment demonstrated that *N-GOQDs* did not exhibit any measurable toxic effects on HaCat keratinocytes across the tested concentrations. Cell viability remained within 94–111% of the untreated control, with low variability, indicating excellent biocompatibility of this formulation. Importantly, the concentrations at which *N-GOQDs* showed no cytotoxicity correspond precisely to the concentrations that produced clear bactericidal activity against all tested Gram-positive and Gram-negative strains. Among the samples, *B,N-GOQDs* also demonstrated low cytotoxicity across all tested concentrations, with cell viability values ranging from 93 to 98% relative to the untreated control. Even at the highest concentration (500 μg/mL), viability remained above 93%, indicating that B,N-GOQDs exhibit favorable safety characteristics for potential biomedical applications. In contrast, *Ph,B,N-GOQDs* showed the greatest cytotoxic effect, with viability decreasing to about 82%.

**Figure 6:**
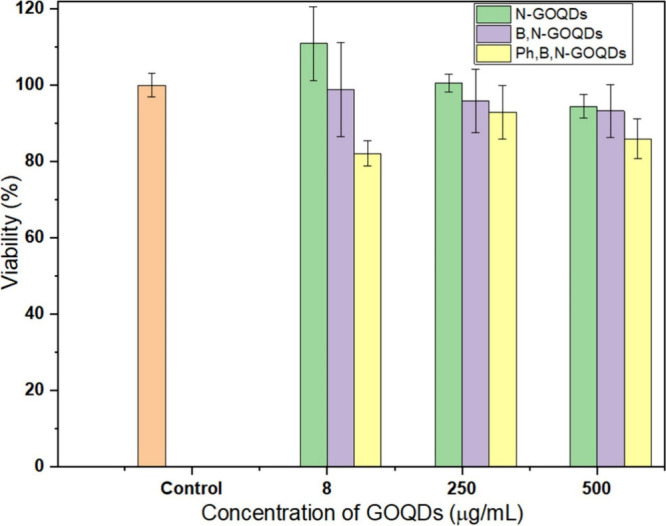
Viability of HaCaT after 24 h of exposure at GOQDs concentrations ranging from 8 to 500 mg/mL. Cell viability was expressed as a percentage relative to the control (100%).

This alignment between high antimicrobial efficacy and a lack of mammalian cytotoxicity is a particularly advantageous characteristic of *N-GOQDs*. It suggests a desirable selectivity for bacterial cells, likely arising from specific physicochemical features, such as optimized surface functionalization or defect structures, that promote antibacterial mechanisms (e.g., membrane disruption and ROS generation)^1^ while remaining benign to human keratinocytes.

Thus, *N-GOQDs* represent the most promising fraction among the tested materials, combining broad-spectrum antibacterial activity with excellent cytocompatibility, making them strong candidates for biomedical applications such as wound dressings, antimicrobial coatings, or other topical formulations, where safety toward human skin cells is essential.

## 4 Conclusions

Comprehensive physicochemical characterization revealed that heteroatom doping substantially influences both the chemical structure and electronic properties of GOQDs. XPS analysis demonstrated effective nitrogen incorporation into the graphenic lattice in all doped GOQDs, primarily in pyrrolic and graphitic forms, whereas the introduction of boron through boric acid or 3-aminophenylboronic acid predominantly affected edge functionalities. In B,N-GOQDs, boron mainly formed oxygen-coordinated species, moderating oxidative cutting and favoring higher graphitic nitrogen content, while in Ph,B,N-GOQDs, organoboron-derived fragments promoted stronger disruption of the sp^2^ network and enrichment of amino-functionalized edge states. These compositional differences were consistent with the observed variations in particle size, morphology, and optical behavior. Optical spectroscopy confirmed that heteroatom composition governs both absorption and photoluminescence characteristics of doped GOQDs. A progressive blue shift of emission maxima from N-GOQDs to B,N-GOQDs and Ph,B,N-GOQDs, as well as increased excitation-dependent photoluminescence in boron-containing samples, reflects changes in the nature and distribution of emissive states and reduction of the lateral size of the doped nanoparticles from 8 to 10 nm for N-GOQDs to 6–8 nm for B,N-GOQDs and further to 4–6 nm for Ph,B,N-GOQDs. Biological evaluation revealed a pronounced dependence of antibacterial activity on GOQD composition. Among the three tested nanoparticles, N-GOQDs exhibited broad-spectrum bactericidal activity against all ESKAPE­(E) panels, with particularly low MBC against *S. aureus*. In contrast, B,N-GOQDs and Ph,B,N-GOQDs showed bacteriostatic activity against *S. aureus*. Cytotoxicity assays using HaCaT keratinocytes demonstrated that N-GOQDs combine potent antibacterial performance with excellent cytocompatibility, achieving bacterial eradication at concentrations that remain entirely nontoxic to human skin cells.

## Supplementary Material



## Footnotes

a CDs can have much higher loading of nitrogen.

b The calculation is based on elemental analysis of nanoparticles (Table 1) and the distribution of nitrogen between pyrrolic (N-5) and graphitic (N-Q) forms.

## Notes

Digital photographs of the Petri dishes documenting the antibacterial activity of the GOQDs against the ESKAPE­(E) pathogens during the analysis (PDF)
