# Supplementary material for: Nitrogen and Boron Co-Doped Graphene Oxide Quantum Dots: Top-Down Fabrication, Comprehensive Characterization and Antibacterial Activity Against Multidrug-Resistant ESKAPE Pathogens
Source: ACS Omega. 2026 May 6;11(19):28898–908. doi: 10.1021/acsomega.6c01888 (PMC13191492; doi:10.1021/acsomega.6c01888)
Supplement: Supplementary file 1 [file ao6c01888_si_001.pdf]

Nitrogen and Boron Co-Doped Graphene Oxide Quantum Dots: Top-Down Fabrication, Comprehensive Characterization and Antibacterial Activity Against Multidrug-Resistant ESKAPE Pathogens

Albina Mikhraliieva[1,2], *Olga Bragina*[2], *Yutao Xing*[3], *Yevgen Karpichev*[2*], *Volodymyr Zaitsev*[1]*

[1]Department of Chemistry, Pontifical Catholic University of Rio de Janeiro, Marquês de Sao Vicente Street, 225, Rio de Janeiro, RJ, 22451-900 Brazil

[2]Department of Chemistry and Biotechnology, Tallinn University of Technology, 15 Akadeemia Rd., 12618, Tallinn, Estonia

[3]Laboratório de Microscopia Eletrônica de Alta Resolução, Centro de Caracterização Avançada para a Indústria de Petróleo (LaMAR/CAIPE), Universidade Federal Fluminense, Niterói, RJ, 24210-346 Brazil

Figure S1. The colony imaging of *S. aureus* (a), *E.faecium* (b), *A.baumannii* and *P.aeruginosa* (c), *K.pneumoniae* and *E.cloacae* (d), *E. coli* (e) on the agar plates with N-GOQDs, B,N-GOQDs, Ph,B,N-GOQDs at varied concentrations 500 – 4 μg/mL.

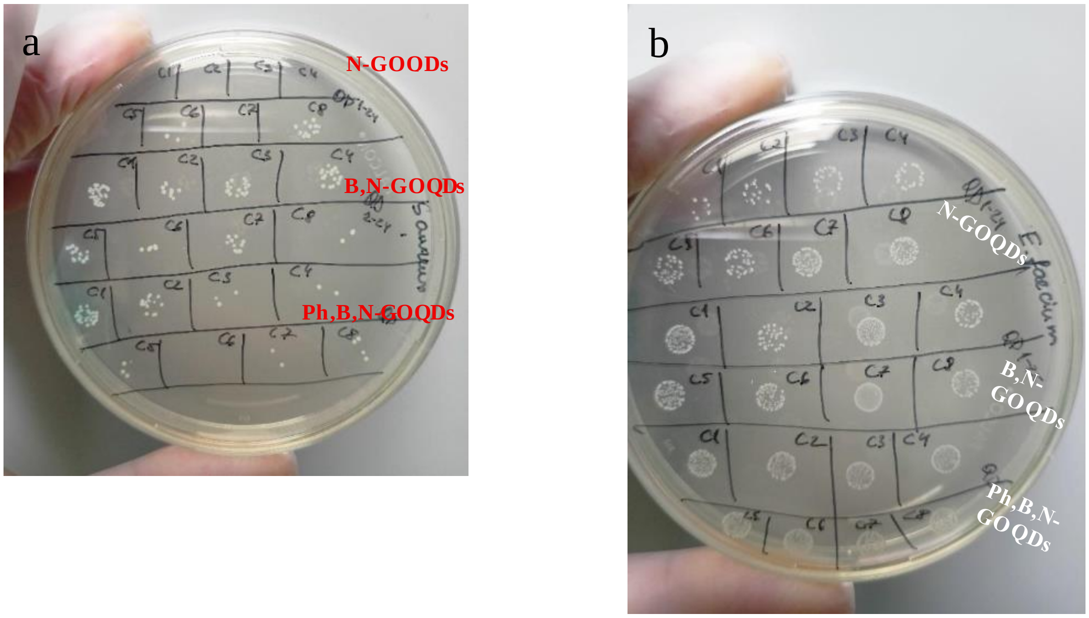

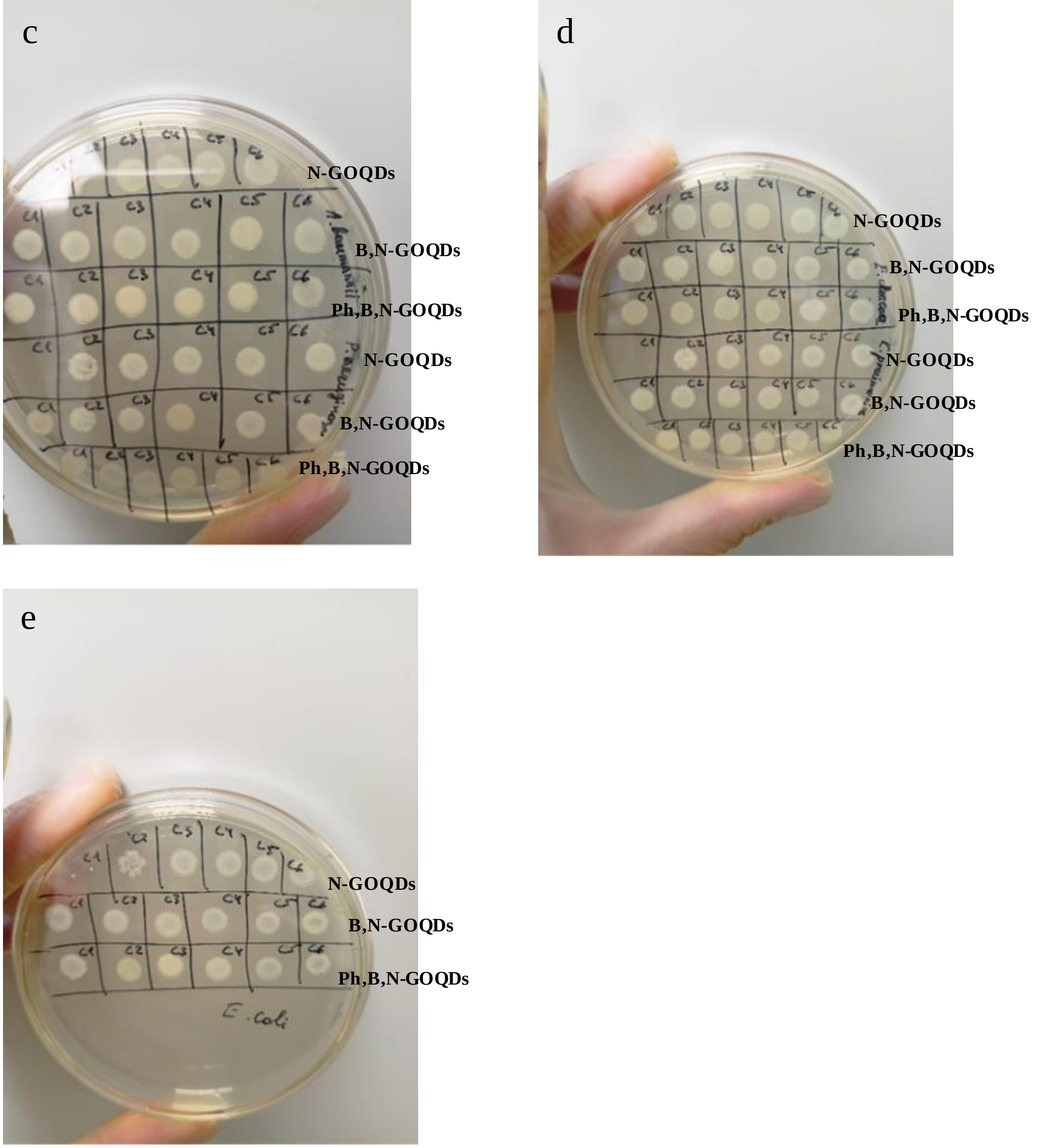
c
N-GOQDs
B,N-GOQDs
Ph,B,N-GOQDs
N-GOQDs
B,N-GOQDs
Ph,B,N-GOQDs
d
N-GOQDs
B,N-GOQDs
Ph,B,N-GOQDs
N-GOQDs
B,N-GOQDs
Ph,B,N-GOQDs
e
N-GOQDs
B,N-GOQDs
Ph,B,N-GOQDs
E. coli
